# Supplementary material for: Electron correlation driven non-adiabatic relaxation in molecules excited by an ultrashort extreme ultraviolet pulse
Source: Nat Commun. 2019 Jan 18;10:337. doi: 10.1038/s41467-018-08131-8 (PMC6338739; doi:10.1038/s41467-018-08131-8)
Supplement: Supplementary file 1 — Supplementary Information [file 41467_2018_8131_MOESM1_ESM.docx]

**Supplementary Information**

**Electron correlation driven non-adiabatic relaxation in molecules excited by an ultrashort XUV pulse**

A. Marciniak^1^, V. Despré^2^, V. Loriot^1^, G. Karras^1^, M. Hervé^1^, L. Quintard^4^, F. Catoire^4^, C. Joblin^3^, E. Constant^1,4^, A. I. Kuleff^2^ & F. Lépine^1^

*^1^ Institut Lumière Matière, Université Lyon 1, CNRS, UMR 5306, 10 rue Ada Byron, 69622 Villeurbanne Cedex, France*

*^2^ Theoretische Chemie, PCI, Universität Heidelberg, Im Neuenheimer Feld 229, D-69120 Heidelberg, Germany*

*^3^ IRAP - Université de Toulouse [UPS] / CNRS 9 Av. du Colonel Roche BP 44346 31028 Toulouse cedex 04 France*

*^4^ Université Bordeaux, CEA, CNRS, CELIA, UMR5107, F-33400 Talence, France*

This Supplementary Information contains 2 Supplementary Tables, 8 Supplementary Figures

| 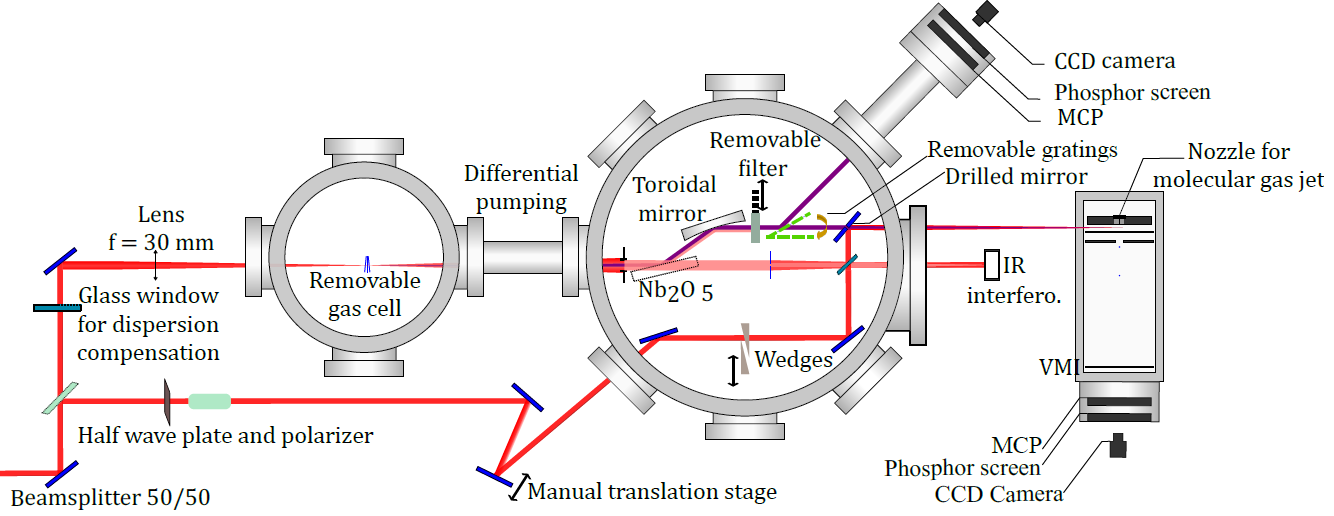 |
| --- |
| **Supplementary Figure 1**: Schematic of the experimental set-up |

| 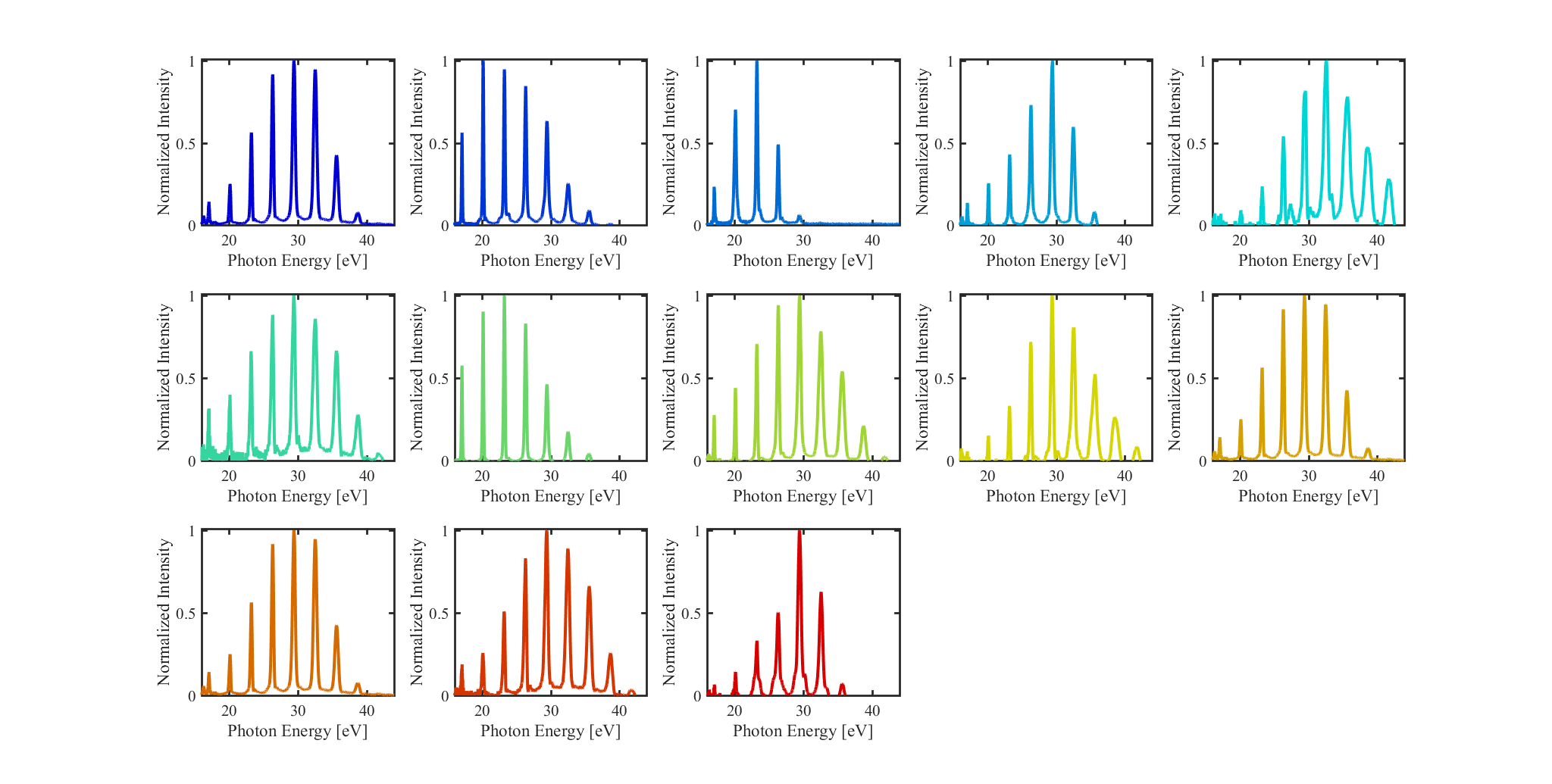 |
| --- |
| **Supplementary Figure 2**: Typical XUV spectra used in the experiment. These spectra have been acquired using an Al filter with a transmission threshold that allows for transmitting only harmonics above 15eV. |

| 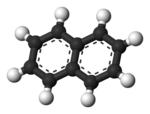 | 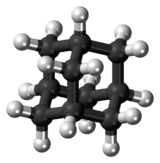 |
| --- | --- |
| **Supplementary Figure 3**: (left) Naphtalene (C_10_H_8_) molecular planar structure. (right) Adamantane (C_10_H_16_) molecular 3D structure. | |

| 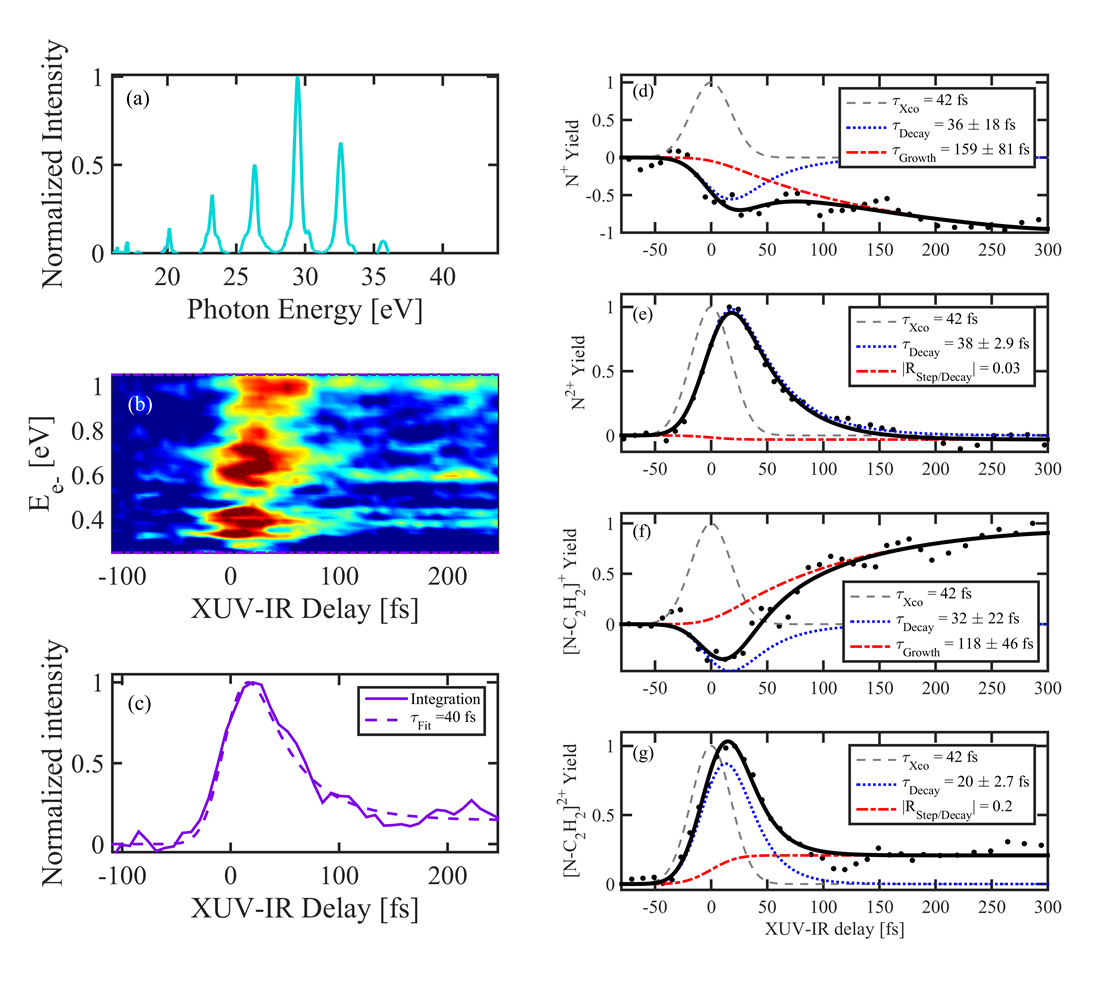 |
| --- |
| **Supplementary Figure 4: a** HHG spectrum used in the measurement, **b** Transient two-color photoelectron map measured under the same experimental conditions as of (a), **c** The averaged electron dynamics is obtained by integrating the transient photoelectron map over the kinetic energy from 0 to 1 eV and its extracted lifetime is 40 ±7 fs.  Transient ion signal, using the same XUV-IR pump-probe scheme for **d** Naph^+^, **e** Naph^2+^, **f** Naph^+^ after C_2_H_2_ loss, and **g** Naph^2+^ after C_2_H_2_ loss. The overall extracted decay time is 38 ± 3 fs. |

|  |  |
| --- | --- |
|  |  |
| 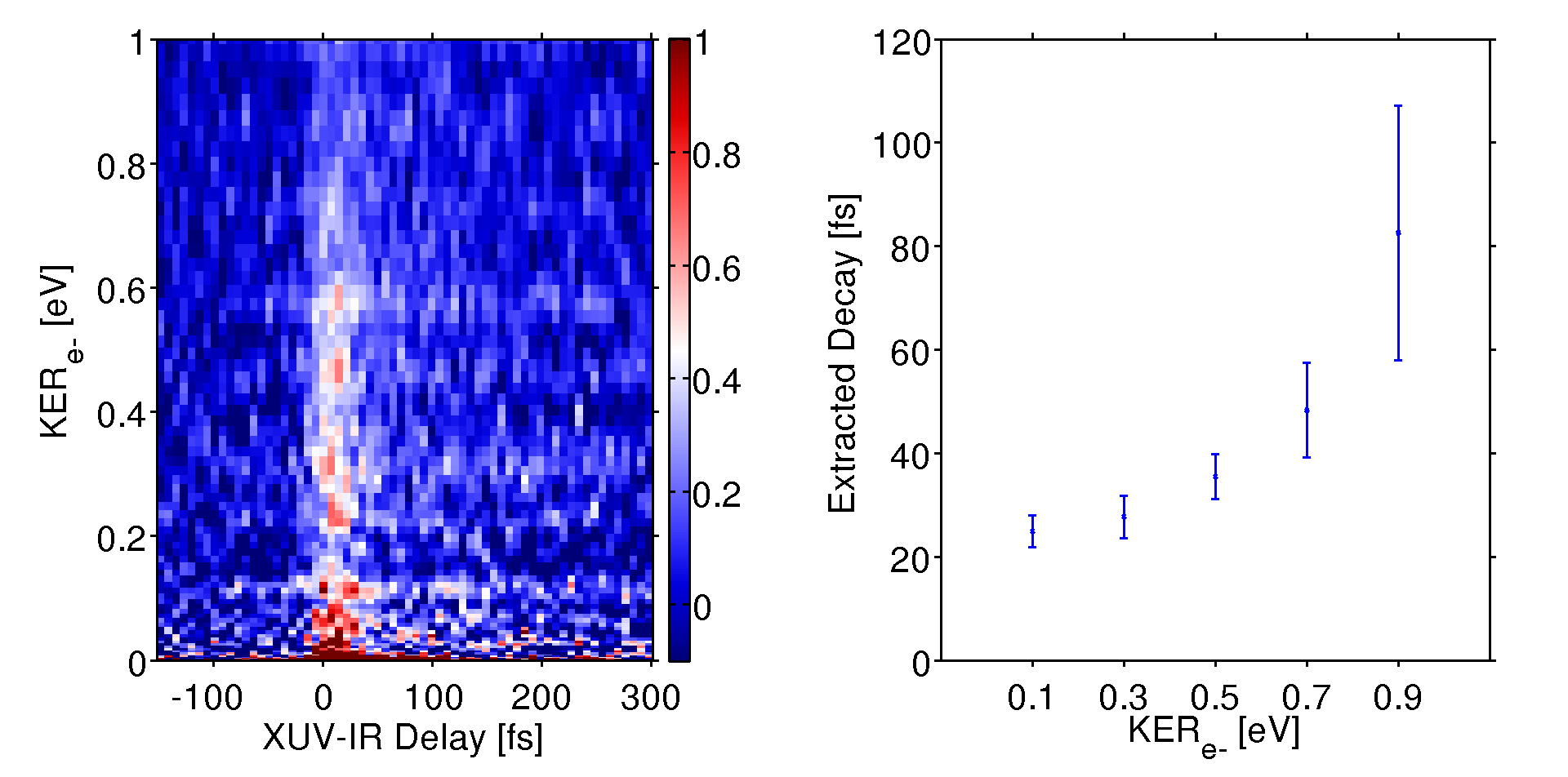 | |
| **Supplementary Figure 5**. **a** Two-color time-dependent electron kinetic energy spectrum obtained in the case of Adamantane. **b** Decay time as a function of the electron kinetic energy for Adamantane. Like in the case of Naphthalene, the decay time globally increases with the electron kinetic energy. | |

| *Supplementary Table 1: States included in the vibronic-coupling Hamiltonian.*   \| number \| symmetry \| Energy [eV] \| Spectral intensity [%] \| \| --- \| --- \| --- \| --- \| \| 1 \| 1b_2u_ \| 19.02 \| 4.2 \| \| 2 \| 1b_1u_ \| 19.1 \| 1 \| \| 3 \| 1a_g_ \| 19.14 \| 1.8 \| \| 4 \| 2b_1u_ \| 19.26 \| 7.5 \| \| 5 \| 3b_1u_ \| 19.34 \| 7.9 \| \| 6 \| 2b_2u_ \| 19.37 \| 1 \| \| 7 \| 2a_g_ \| 19.42 \| 3.3 \| \| 8 \| 3b_2u_ \| 19.44 \| 1.9 \| \| 9 \| 4b_1u_ \| 19.52 \| 4 \| \| 10 \| 3a_g_ \| 19.54 \| 39 \| \| 11 \| 4b_2u_ \| 19.54 \| 1.1 \| \| 12 \| 4a_g_ \| 19.61 \| 2 \| \| 13 \| 5b_1u_ \| 19.62 \| 2.4 \| \| 14 \| 6b_1u_ \| 19.63 \| 4.5 \| \| 15 \| 7b_1u_ \| 19.74 \| 21.9 \| \| 16 \| 5a_g_ \| 19.75 \| 2.9 \| \| 17 \| 5b_2u_ \| 19.84 \| 6.2 \| \| 18 \| 6b_2u_ \| 19.99 \| 6.1 \| \| 19 \| 8b_1u_ \| 20.02 \| 1.1 \| \| 20 \| 9b_1u_ \| 20.05 \| 4.9 \| \| 21 \| 7b_2u_ \| 20.1 \| 32 \| \| 22 \| 10b_1u_ \| 20.11 \| 1.1 \| \| 23 \| 11b_1u_ \| 20.21 \| 3.4 \|   *Supplementary Table 2: Normal modes included in the vibronic-coupling Hamiltonian.* |  |
| --- | --- | --- | --- | --- | --- | --- | --- | --- | --- | --- | --- | --- | --- | --- | --- | --- | --- | --- | --- | --- | --- | --- | --- | --- | --- | --- | --- | --- | --- | --- | --- | --- | --- | --- | --- | --- | --- | --- | --- | --- | --- | --- | --- | --- | --- | --- | --- | --- | --- | --- | --- | --- | --- | --- | --- | --- | --- | --- | --- | --- | --- | --- | --- | --- | --- | --- | --- | --- | --- | --- | --- | --- | --- | --- | --- | --- | --- | --- | --- | --- | --- | --- | --- | --- | --- | --- | --- | --- | --- | --- | --- | --- | --- | --- | --- | --- | --- |

| Symmetry | Frequency [cm^-1^] |
| --- | --- |
| a_g_ | 514.30 |
| a_g_ | 771.15 |
| a_g_ | 1050.83 |
| a_g_ | 1170.08 |
| a_g_ | 1458.28 |
| a_g_ | 1490.03 |
| a_g_ | 1624.66 |
| b_1u_ | 355.71 |
| b_1u_ | 802.70 |
| b_1u_ | 1138.49 |
| b_1u_ | 1276.94 |
| b_1u_ | 1406.41 |
| b_1u_ | 1637.04 |
| b_2u_ | 618.03 |
| b_2u_ | 1042.13 |
| b_2u_ | 1169.70 |
| b_2u_ | 1254.82 |
| b_2u_ | 1494.72 |
| b_2u_ | 1561.41 |
| b_3g_ | 505.72 |
| b_3g_ | 930.99 |
| b_3g_ | 1160.98 |
| b_3g_ | 1254.73 |
| b_3g_ | 1484.04 |
| b_3g_ | 1688.56 |

|  |
| --- |
| **Supplementary Figure 6***: Ionization spectrum of the naphthalene molecule computed with the ab initio nd-ADC(3) method. The states populated by the removal of an electron from orbitals 6a_g_, 5b_1u_, and 4b_2u_ are depicted in green, red, and black, respectively. The vibronic-coupling model is constructed from 23 of those states in the range 19.02 – 20.21 eV. This region of the spectrum is highlight by a blue box. The computed double-ionization threshold (IP_2_) is marked by an orange line. Note that the computed IP_2_ is about 1 eV lower than the measured value of 21.5 eV.* |

| *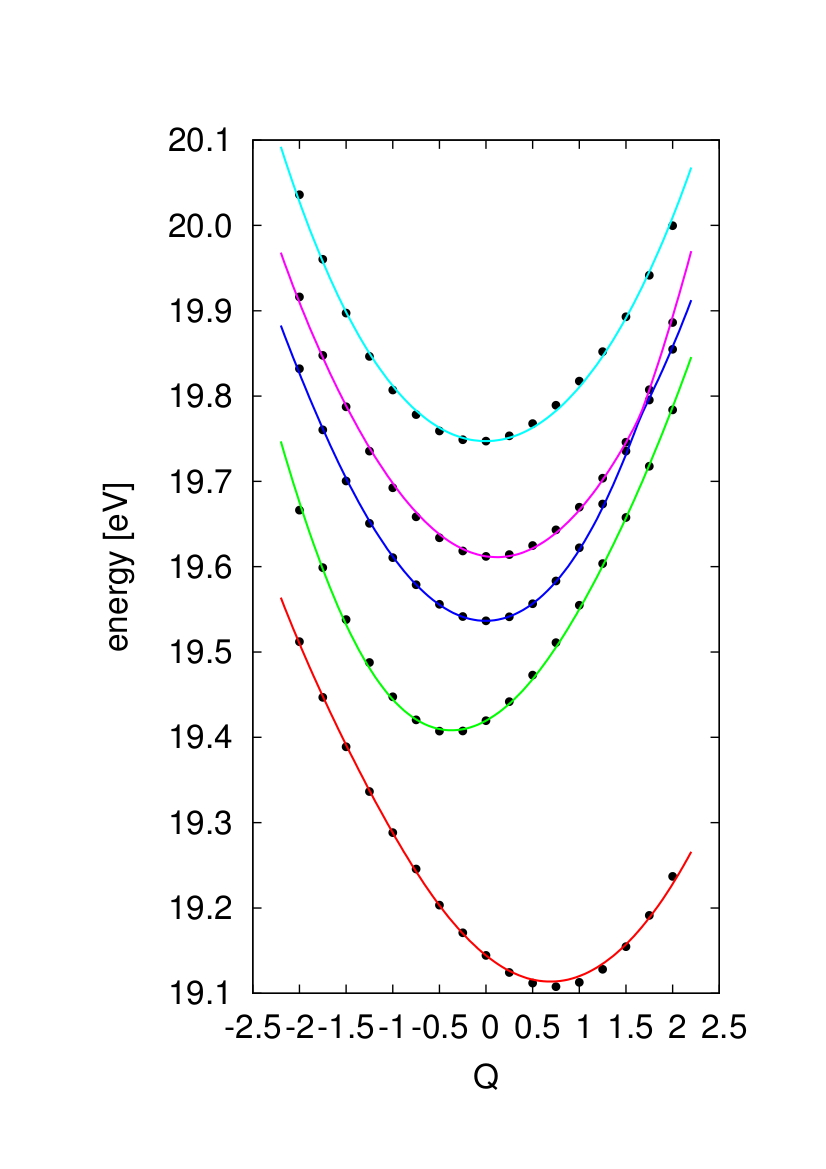* |
| --- |
| **Supplementary Figure 7***: The five states of a_g_ symmetry along the normal vibrational mode of a_g_ symmetry at 1170.08 cm^-1^ computed with the ab initio nd-ADC(3) methods (points) and reproduced by the vibronic-coupling model (solid lines).* |

| *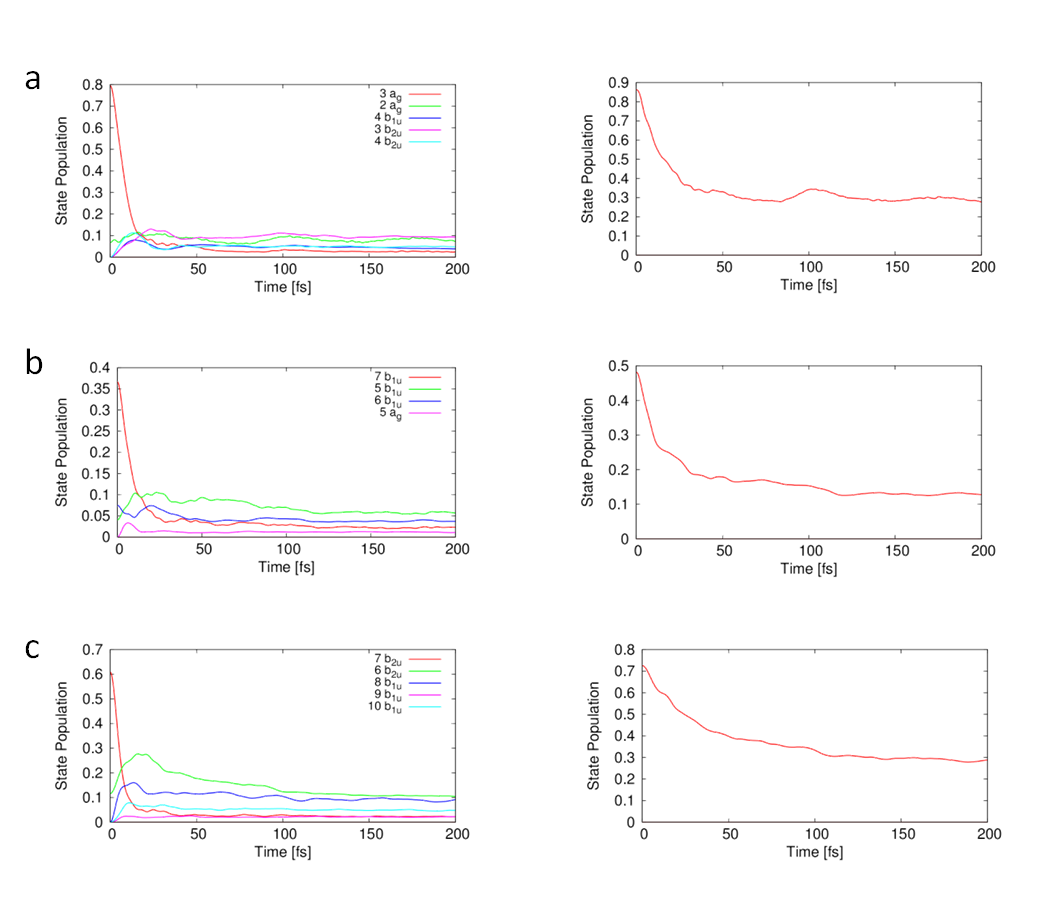* |
| --- |
| **Supplementary Figure 8***: Time-dependent state populations following the initial ionization out of* ***a*** *orbitals 6a_g_,* ***b*** *orbital 5b_1u_ and* ***c*** *orbital 4b_2u_.*  *Left: Individual populations of the states lying within an energy interval of 150 meV.*  *Right : Sum of the populations of all the states in the corresponding energy range.*  *The state labeling see Supplementary Table 1.* |
